# Observing and Studying Extreme Low Pressure Events with Altimetry

**DOI:** 10.3390/s90301306

**Published:** 2009-02-26

**Authors:** Loren Carrère, Françoise Mertz, Joel Dorandeu, Yves Quilfen, Jerome Patoux

**Affiliations:** 1 CLS, 8-10 rue Hermès, 31520 Ramonville St Agne, France; E-mails : fmertz@cls.fr (F.M.); jdorandeu@cls.fr (J.D.); 2 IFREMER, Z.I. Pointe du Diable B.P. 70, 29280 Plouzané, France ; E-mail: yves.quilfen@ifremer.fr (Y.Q.); 3 University of Washington, Box 351640 Seattle WA 98195-1640, USA; E-mail: jerome@atmos.washington.edu (J.P.)

**Keywords:** Altimetry, Detection, Tropical cyclones, Extra-tropical depressions, sea level pressure, barotropic model

## Abstract

The ability of altimetry to detect extreme low pressure events and the relationship between sea level pressure and sea level anomalies during extra-tropical depressions have been investigated. Specific altimeter treatments have been developed for tropical cyclones and applied to obtain a relevant along-track sea surface height (SSH) signal: the case of tropical cyclone Isabel is presented here. The S- and C-band measurements are used because they are less impacted by rain than the Ku-band, and new sea state bias (SSB) and wet troposphere corrections are proposed. More accurate strong altimeter wind speeds are computed thanks to the Young algorithm. Ocean signals not related to atmospheric pressure can be removed with accuracy, even within a Near Real Time context, by removing the maps of sea level anomaly (SLA) provided by SSALTO/Duacs. In the case of Extra-Tropical Depressions, the classical altimeter processing can be used. Ocean signal not related to atmospheric pressure is along-track filtered. The sea level pressure (SLP)-SLA relationship is investigated for the North Atlantic, North Pacific and Indian oceans; three regression models are proposed allowing restoring an altimeter SLP with a mean error of 5 hPa if compared to ECMWF or buoys SLP. The analysis of barotropic simulation outputs points out the regional variability of the SLP/Model Sea Level relationship and the wind effects.

## 1. Introduction

At present, the observation of extreme events such as tropical cyclones (TCs) and extra-tropical depressions (ETDs) through satellite measurements is mainly performed either through visualization of large cyclonic cloudy areas and infrared temperature data (METEOSAT; POES, GOES and DMSP satellites), or via wind field measurements by the Special Sensor Microwave/Imager (SSM/I) or scatterometers (SeaWinds on QuikSCAT [1]; ERS, ADEOS and METOP satellites, [2]). Most altimeter-based studies usually focus on ocean dynamics: by locating warm ocean patterns (such as the Loop Current in the Gulf of Mexico), altimeter measurements allow the detection of ocean heat content anomalies which can be associated with the sudden intensification of tropical cyclones [3,4]. The direct observation of very low pressure systems by radar altimeters has not been investigated yet.

Altimeters (ERS-2, ENVISAT, TOPEX/Poseidon, Jason-1, GFO) provide global sea surface height (SSH) measurements of the ocean under nearly all weather conditions, with the exception of periods of extremely heavy rain, which sometimes occur in hurricanes. The global SSH error for Jason-1 (J1) is estimated to 3.9 cm in normal meteorological conditions [5]. Radar altimeters thus have some potential for determining storm surge heights when flying over the storms. Now, three satellites (J1, ENVISAT, ERS-2) fly together, thus deeply improving the global temporal and spatial altimeter coverage.

The Inverse Barometer (IB) response has been extensively studied for normal meteorological conditions [6–10]; but it remains uncertain that there exists a significant Sea Level Pressure/Sea Level Anomaly (SLP/SLA) correlation during storms and hurricanes, which are generally characterized by heavy rains, high sea states and strong winds.

Indeed, the ocean response to tropical cyclone surface forcing is a complex interaction between baroclinic and barotropic motions that re-distribute energy in the ocean during and after these strong forcing events. This response has been characterized as a predominately baroclinic phenomenon associated with the isopycnal displacements in the thermocline and the excitation of near-inertial three dimensional oscillations. A secondary component is the barotropic response associated with the sea surface depression of several tenths of a cm in geostrophic balance with a cyclonically rotating current field [11,12].

**Figure 1.**
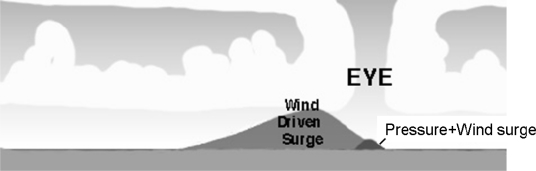
Localization of the storm surge (http://www.aoml.noaa.gov/phod/cyclone).

The inverse barometer effect is balanced by the surface Ekman divergence in the eye of the storm (pressure+wind induced surge on Figure 1). Most (> 85 %) of the storm surge is caused by winds pushing the ocean surface ahead of the storm on the right side of the track in the Northern hemisphere and left side in the Southern hemisphere [11,12].

In general, the strongest winds in a hurricane are found on the right side of the storm (Northern hemisphere) because the motion of the hurricane adds to its swirling winds. Since the surface pressure gradient (from the tropical cyclone centre to the environmental conditions) determines the wind strength, the central pressure indirectly does indicate the height of the storm surges, but not directly.

The aim of this paper is to improve the observation of extreme low pressure events with altimetry and to investigate the relationship between atmospheric SLP and the SLA measurements during such extreme conditions. Issues raised are the problem of the lack of altimeter data due to measurement corruption by rain and to the low accuracy of the different geophysical/instrumental altimetric corrections during TCs; the filtering of the SLA variability not related to atmospheric pressure; and the availability of accurate SLP fields.

The paper is organised as follows: after the introduction, the second part describes the database and the methodology; the third one is dedicated to the detection of tropical cyclones and describes the specific treatments developed for altimeter data; the fourth part gives results about the analysis of ETDs cases and more precisely on the possibility of retrieving a SLP signal from altimeter measurements during ETDs. The last part gives a complementary analysis of the SLP-SLA relationship in the case of ETDs from barotropic model outputs (MOG2D, [1]).

## 2. Database and Methodology

### 2.1. Database

The 2003/2004 time period has been chosen for the analysis because it is covered by several independent databases:

- the ENVISAT, Topex/Poseidon and Jason-1 altimeter missions;
- an extensive observing network deployed in the Atlantic ocean by the National Oceanic and Atmospheric Administration (NOAA). The NOAA hosts the National Hurricane Center (NHC) and the Hurricane Research Division (HRD), which has defined an experimental wind analysis tool to provide regular high-resolution wind fields for tropical cyclones ([13]; http://www.solar.ifa.hawaii.edu/Tropical/tropical.html). This database gives an extensive list of tropical storms which have occurred on all ocean basins, with information on the track of the storm and estimates of the maximum sustain winds, wind gusts and the minimum central pressure. However these estimates give a measure of the storm’s intensity but not of the wind or SLP field which can be easily compared with the altimeter ground track measurements;
- a collocated JASON/buoy database: buoy data include the NDBC network, data available via Météo-France, and the TAO array;
- the ECMWF pressure analyses at 0.5 degree-6 hour resolution;
- the QuikSCAT scatterometer wind measurements; QuikSCAT winds have been assimilated into the ECMWF Numerical Weather Prediction (NWP) model since 2002.

The ECWMF global pressure fields are used to provide long time series of surface pressure with global space/time coverage. However, in this study, we are mostly interested in low and very low pressure systems. In such conditions, NWP models such as ECMWF suffer from limitations related to their coarse space and time resolution, to very few assimilated SLP measurements (aside from those of ships of opportunity limited to the ships’main tracks), and the fact that the dense scatterometer winds are severely under-sampled when assimilated in the NWP. However, we can derive SLP fields from scatterometer wind measurements using an atmospheric planetary boundary layer (PBL) model [14,15]. These QuikSCAT-derived SLP fields have the advantage of retaining the fine scale structures present in the QuikSCAT wind fields. These SLP fields, although accurate for ETD cases, are less accurate for TC situations because the QuikSCAT measurements are often contaminated by rain [16]. This last database has been constituted within this study and will help validating the altimeter-based pressure signal during ETDs (section 4.2).

The SLA derived from altimetry has the following formulation:

(1a) SLA=Orbit−Range−∑Corrections−MSS_CLS

where Σ Corrections = Sea State Bias + Radiometer wet tropospheric correction + Ionospheric correction + GOT2000 ocean tide + Solid earth tide + Polar tide [17,18]. The IB and dry tropospheric corrections are not applied because they are correlated to SLP:

(1b) $$\text{IB}=-0.9948*(\text{SLP}-\bar{\text{SLP}})\;\;\text{in cm}$$

(1c) DryTropo=−2.227*SLP*(1+0.0026*cos(2φ))  in mm [18]

where SLP is the atmospheric pressure (in hPa), $\bar{\mathrm{SLP}}$ is the instantaneous mean of SLP over global ocean, and φ is the latitude. The scale factor 0.9948 is based on the empirical value [19] of the IB at mid latitudes. Several studies have shown the zonal dependence of this coefficient, from about 0.9 cm/hPa at high latitudes to −0.5 cm/hPa at the Equator [9], with a strong space variability due to wind effects and also to some dynamic response to pressure forcing. For the present study, the SLA fields have been computed by subtracting a Mean Sea Surface field MSS_CLS, [20]), to reduce the cross-track geoid’s errors [7,9,21].

**Figure 2.**
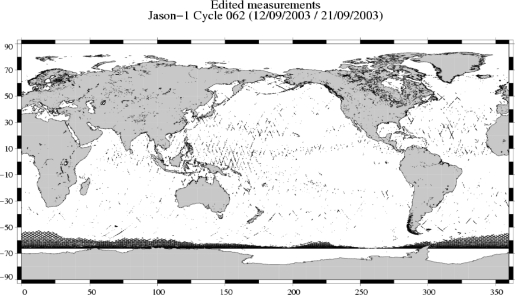
Edited measurements on Jason-1 cycle 062, for ocean applications.

Altimeter data are usually selected using thresholds on the most relevant parameters characterizing the altimeter and radiometer measurement quality. This editing procedure thus allows the selection of useful altimeter datasets for most applications and ocean studies (altimeter Validated Database or VD). However, in the present study, the phenomena of interest can be outside the validity domain conventionally defined, because of extreme conditions such as heavy rain, high sea state and strong wind. Figure 2 shows the location of Jason-1 cycle 062 measurements edited by conventional editing procedures [17]. It shows that, except for rejected measurements over sea ice, altimeter measurements are mainly edited in the warm pool and wet areas because of waveform contamination by rain. Other areas of strong sea state (high waves) are also present. One issue of the study was thus to modify/remove the selection procedure in order to keep enough altimeter data in very low pressure condition.

### 2.2. Global Methodology

The methodology consists in first computing specific SLP and SLA storm databases, respectively for TCs and ETDs. Tropical cyclones and extra tropical storms are treated separately: ETDs are frequent large scale systems, while TCs are more occasional phenomena occurring over very short distances, with great evolution speeds and particularly severe wind and rain conditions. In the TC cases, altimetry measurements are found to be severely corrupted.

Extreme events are localized on the NHC or ECMWF databases (resp. for TCs and ETDs) based on the following criteria:

Wind speed > 17 m s^−1^ for TCs, which is the official threshold for detecting tropical storms

(2) $$\text{DP}=\text{SLP}-\bar{\mathrm{SLP}}<-10\;\text{hPA}\;\text{for ETDs}$$

where $\bar{\mathrm{SLP}}$ is the instantaneous mean global ECMWF SLP.

The low pressure events are placed with the altimeter measurements while screening the pressure or wind speed values within the along-track altimeter databases. With the selection criterion of DP lower that −10 hPa, the typical length of the ETDs detected is greater than ∼1,000–1,500 km. If considering SLP from ECMWF data, as well as from a PBL model applied to QuikScat data, the typical scale of variability of such atmospheric events is between several hundreds of km and about 2,500 km.

To compute the corresponding along-track SLA from altimeters, the conventional validated altimeter databases and corrections are used for ETDs [17], while specific dedicated processing and corrections (described in section 3) are defined for TCs.

In order to study the impact of the pressure forcing on the sea level during extreme events and to improve the SLP-SLA correlation, it is crucial to remove from the SLA signal the ocean variability not related to atmospheric pressure; these other oceanic signals are the mesoscale variability (scales of ∼50–200 km with periods of one to a few months) and the steric variations (seasonal time scale and basin scales; [22]).To that end, several filtering methods have been tested:

- Along-track low-pass spatial filtering with different cutoffs for ETDs and for TCs;
- Removing SLA maps: because the oceanic variability is mainly at low frequencies, one possible filtering method is to remove the low frequency signals by using existing SLA maps (MSLA). These MSLA are routinely produced by SSALTO/Duacs ([18]) with an objective analysis method that combines altimeter missions in both near real time (NRT) and delayed mode (OI, [23]). They are thus optimal observations of the ocean variability by altimeters. In this study, along-track SLA can be corrected with a map of SLA derived from past SLA data (e.g., a map representing the sea level one week before the low pressure event), or using a map recomputed without taking into account the cyclone area.

The quantitative impact of the different filtering methods has been evaluated via the computation of along-track ECMWF DP-filtered SLA correlations: for each storm case, we consider 215 altimeter measurements at 1Hz frequency for ETDs (∼1,500 km), and 100 measurements for TCs (∼700 km).

For ETDs, several filtering with wavelengths between 700 km and 1,500 km have been investigated: a short 700 km cutoff generally does not allow removing the mesoscale variability of the SLA, while on the other hand, the 1,500 km filter can smooth out some pressure induced signal when the scale of the phenomenon is shorter. The best choice should be to adapt the filter wavelength to the scale of the event; however this is not feasible for a systematic analysis and within a real time processing context. The 1,500 km cutoff wavelength has been applied here because it gives the better SLP/filtered SLA correlations on the wide panels of ETDs studied.

For TCs, the MSLA filtering gives better results due to the small scale of the phenomena and to their rapid evolution; this filtering method is very robust even in a real-time processing context. Note that the MSLA filtering is not adapted to ETDs due to their larger spatial scale and to their higher frequency of occurrence.

To study the relationship between the SLP and SLA signals, statistical correlation and regression analyses have been performed on a wide number of storms (during year 2003; cf. Sections 4 and 5). Following the IB approximation used for normal meteorological conditions, a linear regression model between SLP and SLA has been investigated for extreme weather conditions:

(3) DP (hPa)=A*SLA (cm)+B

SLA is the along-track filtered Sea Level Anomaly, and DP is the pressure difference.

As this relation can vary spatially (due to wind effects or pressure enhanced effects in the eye of the storm …), we focused the analysis on the fiercest area of the storm, which is defined as the 16 consecutive along track points with the highest pressure drop (16 altimeter points along the track correspond to ∼100 km, for each storm); all the storms detected on a given basin are considered in a same database. The results are thus mean correlation/regression coefficients for each ocean basin studied (Atlantic, Pacific and Indian): in this case, there is no regional variability taken into account at scales lower than the basin scale.

To investigate the regional variability of the SLP/MOG2D sea level relation (cf. Section 5), correlations and regressions have been performed on the 6-hours ECMWF and model maps, considering for each one degree box all cases with DP < −10hPa in the 2003 period.

Note that as the dry tropospheric correction has not been applied to SLA, this correction is implicitly included in the resulting regression model of equation (3). Following equation (1c) and since the variability of the dry tropospheric correction is about five times smaller than the standard IB, this would lead to IB-like coefficient of about −1.12 cm/hPa at high latitudes (or A = −0.89 hPa/cm for equation (3)).

The regression models will be validated using independent datasets in section 0.2: ECMWF pressure during different time periods from the analysis period, QuikSCAT-derived SLP, and in situ data.

## 3. Detection of Tropical Cyclones

**Figure 3.**
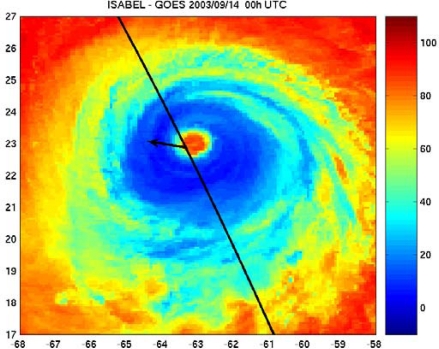
GOES infrared brightness temperatures measured in TC Isabel and the Jason altimeter ground track in black. The arrow indicates the direction of the cyclone motion.

Altimeter dual-frequency measurements can provide valuable information for tropical cyclones analysis and forecasting. Indeed, although limited by their dimensional sampling for operational use, the dual-frequency capability makes altimeters a unique satellite-borne sensor performing measurements of key surface parameters in a consistent way, i.e. surface winds, sea state, and rainfall rate. It is especially true where and when no aircraft measurements are available and when the classical Dvorak intensity analysis may be less accurate (at night, or when the cyclone eye is partially or totally obscured by clouds). This is illustrated in Figure 3, which displays the infrared GOES images the closest in time to the Jason altimeter track intersecting TC Isabel. These altimeter data were unique at that night time. Careful analysis of the altimeter retrieved wind speed and associated sea state could certainly help the intensity analysis performed from the GOES image.

For studying tropical cyclones, the first issue is the lack of reference surface pressure data and wind fields. The NWP surface fields are less accurate for TCs because of the model resolution, the limitations in the physics and parameterizations, and the lack of observations to assimilate into the model. The HRD wind analysis benefit from various sources of observations (buoys, aircraft, satellite) but, although useful, it is a limited data-set which accuracy is strongly affected by the scarcity of surface data [13]. Finally, the ECMWF/QuikSCAT merged SLP fields presented in the previous section are hardly usable in TCs situations because the QuikSCAT measurements are heavily contaminated by rain [16].

The second issue is the lack of validated altimeter data during TCs. Due to their short scale and very high propagation speed compared to satellite inter-track distances and delay, frequent satellite flying over the eye of a hurricane is unlikely, unless a constellation of altimeters is operated. Moreover, when it does happen, altimeter measurements are severely affected: the measurements are corrupted by rain (Ku-band is very sensitive to rain) and the different geophysical/instrumental altimetric corrections used are not accurate enough for extreme weather.

Specific altimeter data processing is thus needed in order to improve the capability of altimetry to observe TCs and to allow extreme sea state conditions in the database. The points with the ice flag, the land flag and the S-band anomaly set on are still eliminated. The problem of rain contamination is partly fixed using C- or S-band measurements on Jason 1 and Envisat respectively; these bands are less affected by rain than the Ku band, but they are also noisier. SSB and wet Troposphere correction need to be recalculated in this specific extreme weather context.

Note that due to the lack of accurate SLP data and to the heavy altimeter data treatments needed, this part focuses on the SLA signal restitution during TCs and on the detection of such systems from altimetry.

### 3.1. Rain Effects and Computation of New σ_0_ and Wind Speed

Even though the Ku band is corrupted in tropical cyclone cases [2] due to strong attenuation by rain, an expected-Ku band backscatter coefficient (σ_0_) can be recomputed from the σ_0_ at the lower frequency (C-band for Topex and Jason, S-band for Envisat) along with the mean rain-free relationship between the two frequencies (Ku/C and Ku/S) [24]. An iterative algorithm is used to account for the fact that C or S-band are also affected by rain, although the attenuation is small at these frequencies. The Young algorithm [25] and the expected Ku σ_0_ allow the computation of a new wind speed for values over 20 m s^−1^.

**Figure 4.**
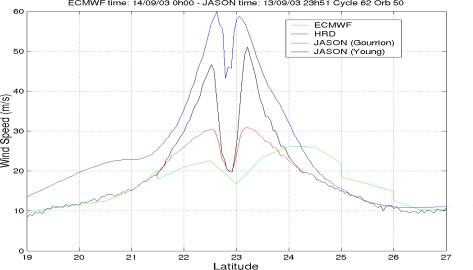
Comparison of different wind speeds for Jason-1 during tropical cyclone Isabel.

This new altimeter wind speed is shown in Figure 4 for TC Isabel: it is stronger than the ECMWF value and closer to the HRD measurements in the fiercest area of the storm. All details about the methodology are given in Quilfen *et al*. [26].

### 3.2. Wet Troposphere Radiometer Correction

For J1 data, the GDR wet tropospheric correction is used. In this case, the algorithm is parametric and uses three channels of the radiometer, including the 18.6 GHz channel which gives information about the sea surface; it can retrieve consistent values of the wet tropospheric correction during extreme events. These values will be used to validate the new wet tropospheric formula developed for Envisat and described hereafter.

**Figure 5.**
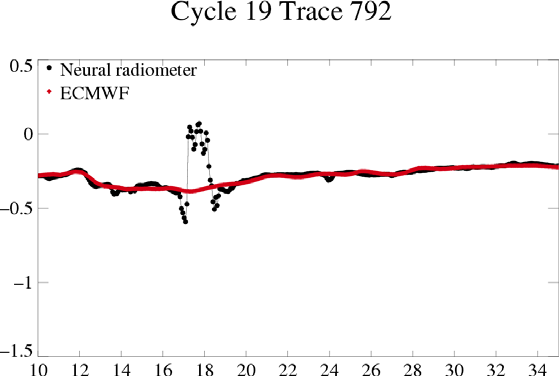
values of the neural radiometer and ECMWF wet troposphere corrections (in m) for Envisat during TC Isabel, in function of latitude (degrees).

The neural wet troposphere correction used for the Envisat data is very noisy and is not formulated for high sea state conditions [27], as shown in Figure 5 for TC Isabel. The ECMWF correction is underestimated in such extreme conditions and cannot be used either.

#### 3.2.1. The Parametric Algorithm for Envisat

**Figure 6.**
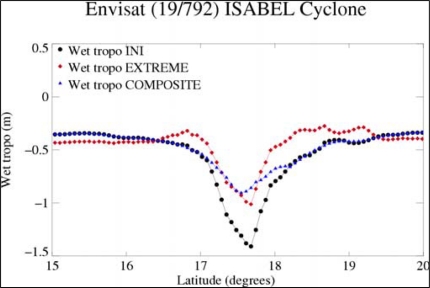
Wet troposphere correction computed for Envisat for TC Isabel (cycle 19-track 792).

The radiometer correction can be deduced from the brightness temperatures and the wind speed or backscatter coefficient in Ku band; this last parameter, wind speed or σ0, gives information about the sea surface. The parametric formula used in the beginning of the distribution of the Envisat GDRs is based on the radiometer brightness temperatures (two channels onboard Envisat: 23.8 and 36.5 GHz) and on the backscatter coefficient (σ0 in Ku-band). It is a multilinear algorithm fitted on the basis of normal surface conditions [28]: the drawback of this initial computation is the overestimation of the correction in extreme conditions where it can reach 1.5 m (black curve on Figure 6).

A new parametric formula has thus been computed, including in the learning database the altimeter winds greater than 25 m.s^−1^ as defined in the previous section (from S-band σ0 and Young algorithm).

(4) $$\begin{array}{l} \text{Wet}\_{\text{tropo}}_{\text{Extreme}}=62.1360\\ -58.1935*\text{LOG}(280-{\text{TB}}_{23.8})\\ +48.1591*\text{LOG}(280-{\text{TB}}_{36.5})\\ -0.236073(\text{WindSpeed}-7) \end{array}$$

where the wind speed is in m.s^−1^, the brightness temperatures (TB) are in K, and the resulting correction is in cm. This new parametric algorithm has a minimum adjustment error of 4cm.

The new correction (red curve) has weaker values during TCs but is not adapted for lower wind speeds around the area of the TCs (around 17°N and 18°N). In order to obtain a more realistic wet tropospheric correction all along the track, the two parametric corrections have been combined into a composite correction (blue curve): if the initial correction is greater than 0.5 m (which is the threshold for normal sea state conditions), the recomputed wet tropospheric correction is taken. A final smoothing avoids any discontinuities between the two corrections.

#### 3.2.2. Validation of the New Parametric Correction

**Figure 7.**
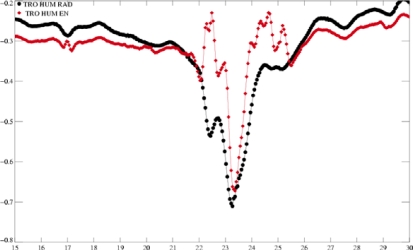
Envisat parametric formula calculated with Jason-1 TBs (red) compared to the radiometric correction (black) for Jason for TC Isabel (J1 track 50/cycle 62). Wet troposphere correction unit is m.

For validation, the new parametric formula obtained for Envisat (Equation 4) has been applied with the Jason-1 TBs and the wind speed recomputed for J1, on J1 track 50/cycle 62 over flying TC Isabel. The first channel is the same for Envisat and J1 (23.8 GHz) but the second one is slightly different (36.5 GHZ for Envisat and 34 GHz for J1). This can lead to a difference in the two algorithms for normal conditions, but the result for TC Isabel (red curve on Figure 7) shows that the formula is appropriate for the higher values of winds and TBs, where the correction reaches 0.7m as for the correction given by J1’s algorithm. Again we notice that the values around the event are not consistent, which points out that this model is not adapted to normal conditions.

#### 3.2.3. The Neural Algorithm

**Figure 8.**
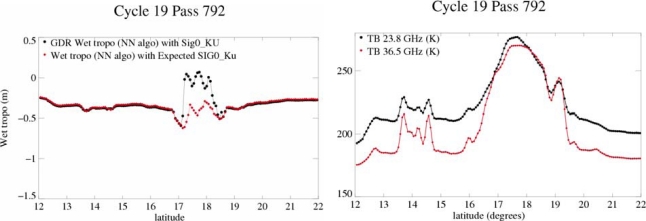
Values of the wet tropospheric corrections computed with Neural Network algorithm with SIG_0_-Ku from GDR (black) and “expected” SIG_0_-Ku (red) (left) and values of TBs (right).

Quartly [29] applied the neural algorithm using the “expected” Ku Sigma0 defined in section 3.1 as input to compute the wet tropospheric radiometer correction. In the case of TC Juan [30], this approach gives a continuously increasing water vapor curve until 90 kg/m^2^ (which corresponds to a 56 cm decrease in the wet tropospheric path delay) in the fiercest area of the storm. But in the case of TC Isabel, the results are not realistic (Figure 8, left): the recomputed correction (red) gives estimations that are within the usual editing criteria (0 and 50 cm), but its along track variability is not continuously decreasing as expected during an extreme event. The inconsistency of the correction is likely due to the stronger conditions in TC Isabel: higher rain and stronger brightness temperatures (Figure 8, right).

### 3.3. SSB Estimation

A new SSB estimation has been computed in S-band for Envisat and for extreme low pressure cases. For normal sea states, the S-band SSB is computed with the non parametric algorithm using the Ku-band SSB [30].

In extreme conditions, the Ku-band is unusable. Moreover, if considering only extreme events (one year of crossover data with altimeter wind speeds over 15 m s^−1^), the number of points is reduced and the NP SSB algorithm is very close to fitting a simple linear model (SSB=A*SWH):

(5) New SSB (Envisat)=−6%*SWH

where SWH is the significant wave height in m. The error of this algorithm is below 1% of SWH for normal meteorological conditions.

This new SSB is very close to the NP SSB for SWH values of 6 m and 20 m s^−1^ wind speed, which shows the continuity between the SSB values in normal and extreme meteorological conditions.

In the same way, a computation of SSB has been made for Jason-1, based on C-band data:

(6) New SSB (J1)=−4.6%*SWH

### 3.4. SLA Noise Reduction for TCs Applications

**Figure 9.**
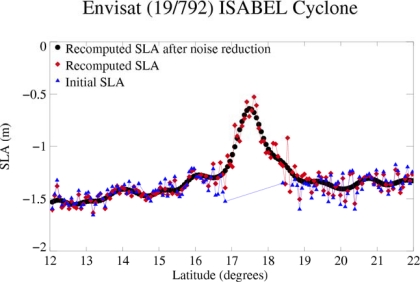
Comparison of initial SLA (blue), recomputed SLA with wet tropospheric and SSB corrections (red) and recomputed SLA with the noise reduction applied (black) in m for Envisat during Tropical Cyclone Isabel.

The C and S bands are less affected by rain than the Ku band, but they are noisier. Table 1 gives the standard deviation of each 1-Hz measurement of the SWH (m), SIGMA-0 (dB) and Range (m) in the two bands for Jason-1 and Envisat. These values are derived from the standard deviation of 20-Hz altimeter measurements used to compute 1-Hz estimations. High noise tends to reduce the correlation between SLA (derived from C- or S-band measurements) and sea level pressure. The along-track filtering of S- and C-band measurements for Envisat and Jason-1 respectively has been tested for several wavelengths. A simple Lanczos filtering ([31]) of 85 km significantly reduces the noise in the SLA computation, as shown on Figure 9 (black curve).

### 3.5. SLA Filtering for TCs Applications

The MSLA filtering removes the ocean signal not related to atmospheric pressure with good accuracy during TCs. Several SLA maps have been computed in order to improve the filtered signal: optimal interpolations (OI, [23]) have been performed using the complete data set with a window of 40 days or removing spatial and temporal areas corresponding to the cyclone. A correlation analysis between SLA-MSLA and SLP has shown close results for the different mapping, confirming the robustness of this filtering method.

Note that the MSLA filtering has been tested in a Near Real Time (NRT) context, using in the OI only the measurements before the day of the cyclone with a decentred window: results are very similar to the off-line MSLAs and show the efficiency of the method and the ability of NRT altimetry to detect such extreme events.

**Figure 10.**
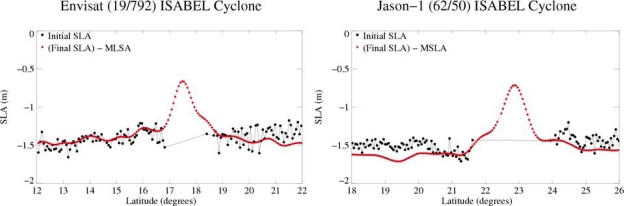
Initial and final SLA computed for Envisat (cycle 19, track 792) and Jason-1 (cycle 62, track 50) during Tropical Cyclone Isabel.

Figure 10 shows the reconstructed signal (SLA-MSLA) using all new dedicated processing (SSB for Jason-1 and Envisat, Wet troposphere for Envisat) for TC Isabel. One gets a very interesting along-track SLA signal within the fiercest area of the storm, showing a storm surge greater than 50 cm: it is consistent with an IB response to a strong low pressure forcing. Notice that, at the present time, no in situ measurement is available to validate quantitatively this new SLA during TCs.

## 4. Retrieving SLP during Extra Tropical Depressions

The relationship between SLP and SLA during extra-tropical storms has been investigated in order to retrieve the surface pressure from altimeter measurements. The ECMWF pressure fields and the altimeter validated database are used. For ETDs, the main issue is to filter out ocean variability not related to atmospheric pressure forcing: an along track low-pass filter has been applied systematically with a wavelength of 1,500 km. A variable wavelength adapted to the size of each depression allowed a better filtering but is hardly usable for a systematic analysis.

### 4.1. SLP-SLA Regression Analysis

The analysis covers all ETDs occurring during year 2003. The altimeter filtered SLA and the ECMWF pressure fields are used to fit the A and B regression coefficients (from Equation 3) through a least-squares method focusing on the fiercest area of the storms. The analysis has been performed separately for three different ocean basins: North Atlantic, North Pacific and Indian Oceans. The best correlations between SLP and filtered SLA have been obtained when excluding coastal areas and strong mesoscale variability areas from the analysis (cf. Table 2). Note that the correlations are strong in the three zones, and the regression coefficients are very stable within a 95 percent confidence level.

A is smaller than the 1hpa/1cm value, because the dry tropospheric correction has not been applied to the SLA (cf. 2.2). Thus the values of A are not very different from the standard IB in the three oceans. During extreme events one would expect that strong winds will impact the sea level, which is not appearing on these regression values: this might be due to the global approach (means on wide basins, unique along-track SLA filtering). A regional analysis will better point out the wind effects (see sections 5.2, 5.3).

The rms error of the regression model (Table 2), if compared to ECMWF SLP in 2004, is 5.2 hPa for the three oceans. As the Saffir-Simpson scale used to classify hurricanes/depressions, defines each category within a pressure increment of 15/25 hPa [32], such observations with an accuracy of 5 hPa or less are valuable. However during ETDs, one can still expect reducing this error while improving the SLA and/or the filtered SLA signals and the regression model (cf. section 5).

### 4.2. Validation of the Altimeter SLP during ETD

Comparisons of the altimeter restored SLP have been performed with different datasets: QuikSCAT-derived SLP, ECMWF SLP and the collocated Jason/buoy product. Buoy data include the NDBC network operated by NOAA and available via internet, data acquired in Europe available through Météo-France, and data from the TAO array operated by PMEL. The criteria for collocation between buoy and altimeter measurements are: maximum time separation of 60 min, maximum spatial separation of 50 km. These databases are available at IFREMER. Only off shore buoys with an estimated local pressure difference greater than 10 hPa have been used for the validation, which reduces the dataset to 162 collocated measurements.

**Figure 11.**
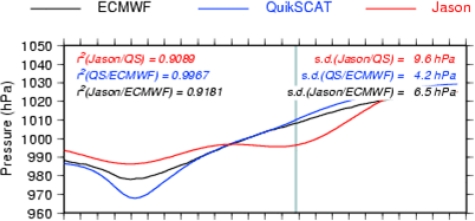
Comparison of the along-track SLP for the ETD of 11/01/2004 2:50 UTC QuikSCAT time. The abscissa shows the along-track distance in km (one stick every 100 km).

Table 3 presents some Jason-QuikSCAT and Jason-ECMWF statistical comparisons: the mean correlation is close to 0.95 for all ocean basins. The percentage of collocations with a correlation lower than 0.8 is close to 10, with significantly lower values in the northern hemisphere. This difference between the northern and southern hemispheres is likely due to the strong variability associated with the ACC, which may pollute the pressure-induced SLA signal in the southern hemisphere. Figure 11 illustrates the problem of the along-track filtering process within strong mesoscale areas (ACC variability): the method cannot properly extract the pressure-induced signal.

**Figure 12.**
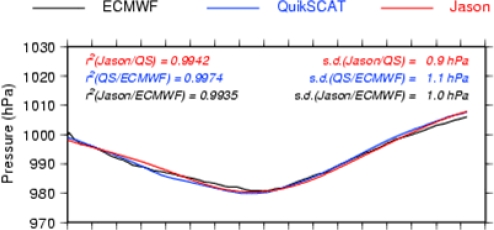
Comparison of the along-track SLP for the ETD of 23/04/2004 21:08 UTC QuikSCAT time. The abscissa shows the along-track distance in km (one stick every 100 km).

**Figure 13.**
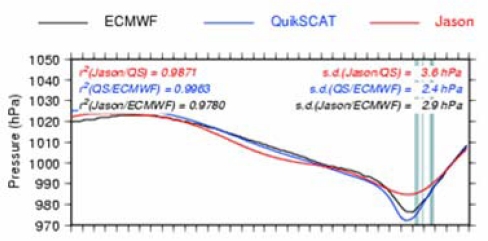
Comparison of the along-track SLP for the ETD of 21/03/2004 5:12 UTC QuikSCAT time. The abscissa shows the along-track distance in km (one stick every 100 km).

When the ETDs are strong and large-scale when compared to the background variability, an accurate pressure signal can be retrieved from altimetric measurements (Figure 12). But the error can be greater for smaller-scale ETDs that might be smoothed out by the large-scale along-track filtering, and due to strong and localised wind effects that are not taken into account within the basin-wide mean regressions (Figure 13).

**Figure 14.**
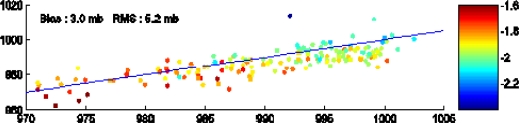
Comparison of the Jason SLP with buoys measurements (abscissa). The colored bar gives the SLA in m.

Figure 14 shows the comparison of the Jason SLP with buoys measurements. The Jason SLP is overestimated by about 3 hPa, which reflects the mean overestimation of the ECMWF SLP used to calibrate the SLP/SLA relationship in low pressure systems. The rms error between Jason and buoys SLP is only 5 hPa. There is a small residual dependency of the error (Jason – buoy SLP) on the sea level anomaly, which means that the regression models could still be improved. It should be noted that a multivariate regression analyses between SLP, filtered SLA and the altimeter wind speed, did not significantly improve the rms error.

## 5. Comparisons with a Dynamical Modeling Approach for ETD

Barotropic simulations are used to further investigate the SLP-SSH relation during mid-latitude storms. MOG2D is a finite elements non-linear gravity wave model using shallow water equations. This global barotropic model allows simulating the high-frequency response of the ocean to atmospheric pressure and wind forcing [6,33]. The model is forced by ECMWF pressure and wind fields. MOG2D outputs only contain, in essence, atmospherically forced signals; if neglecting model errors, the model sea level thus represents the ideal along track filtering and the ideal altimeter measurement of the SLA signal.

### 5.1. SLP/MOG2D Sea Level Regression Analysis

The mean regression between MOG2D sea level and the ECMWF SLP has been performed on all 2003 ETDs (Table 4). Note that the MOG2D sea level does not need to be along-track filtered as it is, in essence, only representative of the ocean response to atmospheric wind and pressure forcing.

As expected, the linear regression coefficient A is greater than 1 hPa/cm in absolute value, reflecting the anticorrelation between the pressure and the wind effects. The global error on the restituted pressure is significantly weaker when considering the MOG2D signal instead of altimeter measurements (Table 2): the improvement reaches more than 30 % for the North Atlantic and North Pacific oceans and represents the minimum gain we could get on the altimeter-based regression models, while improving the filtering of altimeter data from all signals not related to atmospheric forcing. The residual error is due to model errors (physics approximations, grid size, bathymetric errors, atmospheric forcing errors [34,35]), and to the limitation of the global linear regression model. Note that a multivariate and linear regression analysis between SLP, MOG2D sea level and altimeter wind speed, did not significantly improve the performance of the regression model to restore atmospheric pressure.

### 5.2. Regional Variability of the SLP-SLA Relation

**Figure 15.**
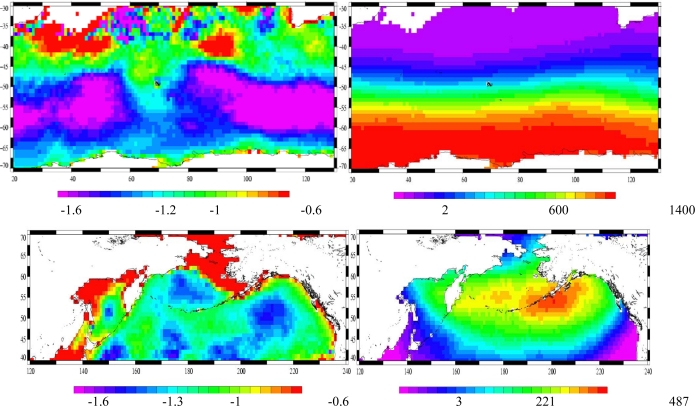
Upper panel is Indian ocean, lower panel is north Pacific ocean. Left: regression coefficient A between SLP and MOG2D sea level (hPa/cm). Right: number of cases with DP < −10hPa.

The spatial variability of the SLP-MOG2D sea-level relation has been investigated: the regression coefficient A is plotted on Figure 15 for the Indian and the north Pacific oceans. As expected, it has a strong spatial variability [9]: A is smaller in absolute value, in coastal areas, due to non-linearities, dissipation, and coastal wind effects [36]. A is greater in absolute value, in deep ocean regions where the ocean has a strong dynamic response to wind forcing [6,37], due to the anticorrelation between the wind and pressure effects; these results are consistent with prior studies on the regional IB effects during normal meteorological conditions [8,9]. Note that north of 45°S in the Austral Indian ocean, the analysis is not significant because of too few extreme cases.

### 5.3. Focus on the ETD of 03/21/2004 5:12 UTC QuikSCAT

**Figure 16.**
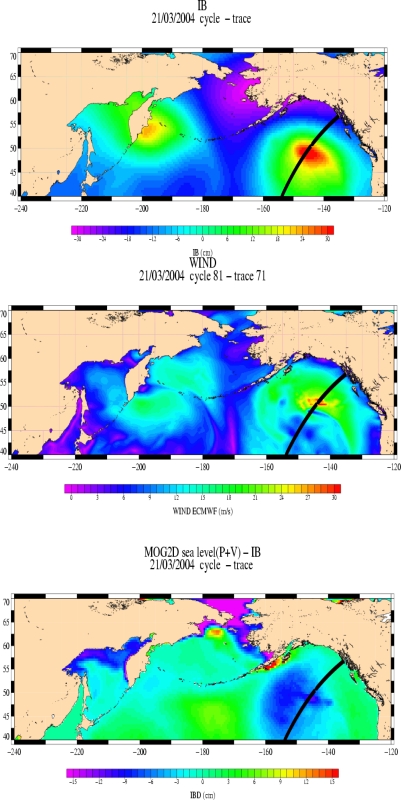
Case of 21/03/2004 over the North Pacific Ocean – J1 cycle 81 – track 71. (a) map of the IB signal at the time of the event, in cm (top panel). (b) map of the ECMWF wind speed in m.s^−1^ (center panel). (c) map of the IBD signal in cm (lower panel).

**Figure 17.**
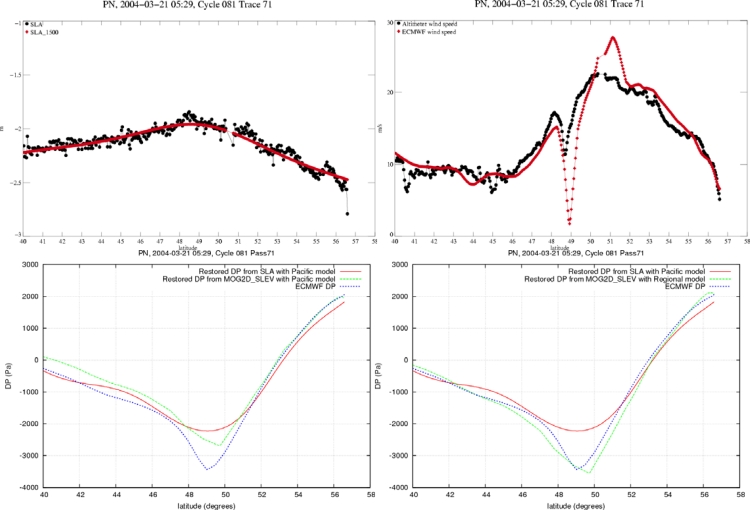
Case of 21/03/2004 over the North Pacific Ocean – J1 cycle 81 – track 71. Along-track plots, abscissa is latitude. (a) SLA in black and filtered SLA in red (cm, upper left panel); (b) ECMWF wind speed in red and altimeter wind speed in black (m.s^−1^, upper right panel); (c) ECMWF DP in black, altimeter-restored DP from global model in red and from barotropic model global analysis in green (Pascals, lower left panel); (d) same as (c) with green line showing DP from the regional regression model (Pascals, lower right panel).

We focus on the ETD of 03/21/2004 in the north-east Pacific Ocean. We compare the restored pressure signals from the SLA based regression analysis on one hand (cf. 4.1) and from the MOG2D sea level regression models on the other hand. In this last case, MOG2D model is forced with ECMWF wind and pressure and then the pressure is estimated from the model sea level (as described in sections 5.1 and 5.2). Figure 16 shows the collocated Jason-1 track on the ECMWF IB signal (a), the ECMWF wind speed map (b), and the IB deviation (IBD) map computed with the MOG2D model (c). Figure 17 shows the along-track unfiltered and the 1500-km low-pass filtered Jason-1 SLA (a), the along-track ECMWF and altimeter wind speed profiles (b), and the along-track SLP profiles (c, and d for regional regression model in green).

This ETD has a small spatial scale (about 500 km) if considering the pressure gradient DP > 10 hPa, with a sharper pattern about 48°–49° North (Figures 16a and 17a). The altimeter and the model wind profiles are in good agreement: the minimum in wind speed is well localized around 48°–49° North, but the ECMWF wind speed gradient is too sharp (Figure 17b).

The local response of the SLA to this forcing is not clear in Figure 17a, because it is of the same order of magnitude as the surrounding mesoscale variability; the 1,500-km along-track filtering applied on SLA for the systematic analysis (cf. 2.2 and 4.1) smoothes out most of this small scale ETD signal (examples Figure 11, Figure 13).

MOG2D simulation clearly shows a local response of the ocean to these forcings (cf. Figure 16c): a small cell of strong IBD signal (IBD = sea level – IB), reaching −12 cm, is localised under the satellite track. This small spatial scale dynamic response is clearly due to a local enhanced response of the ocean to the sharp wind forcing.

The restored DP based on altimeter SLA (from model defined in section 4.1) is widely underestimated and too smoothed likely due to the too large scale filtering applied (red line in Figures 17c–d).

The restored DP based on the MOG2D model (from the model defined in Section 5.1) has a better spatial variability, but its absolute value is still lower than the ECMWF pressure field (cf. green line in Figure 17c). This lower restored pressure could be due to a too weak regression coefficient; indeed, the basin-wide regression coefficient used is smaller in absolute value than the regional coefficient inferred from section 5.2.

If using the regional regression model (A = −1.3 hPa/cm from Section 5.2), the restored SLP is closer to the QuikSCAT pressure field shown in Figure 13 (blue line), although it is still a bit underestimated within the fiercest area of the storm (SLP is plotted in green in Figure 17d).

This local underestimation of the MOG2D-based restored pressure can be explained by the small-scale signal (due to sharp wind forcing) which cannot be well modelled by the too large finite element mesh in this area (grid cells of about 200 km).

This analysis shows the importance of the regional variability of the relationship between sea level and atmospheric pressure as previously shown for normal meteorological conditions ([9]). The along-track filtering of SLA is also a challenge as the filter wavelength should be adapted to the scale of the event but should also be able to deal with the mesoscale signal. Using model outputs gives interesting results although it is still limited by resolution issues and, forcing and model errors.

## 6. Conclusions

Many SLA altimeter measurements during TCs have been restored with a new dedicated altimeter processing using S or C band; specific SSB and wet tropospheric corrections have been computed for extreme events. A quantitative validation of this SLA signal was not possible due to the lack of in situ data during TCs. However these new SLA data are now available for the scientific community for use and validation. A more accurate altimeter wind speed has been computed for extreme winds [26]. For TCs, the MSLA filtering has proven to be very efficient at extracting the atmospherically forced signal, even in a NRT context, showing the ability of altimetry to detect such extreme events.

This study showed that a pressure signal can be retrieved from altimetric measurements during extra-tropical storms with a good correlation between SLP and SLA (>0.8). The regression model error is about 5hPa; mesoscale variability areas as well as intense and localised wind effects are a strong error source. The along-track filtering of SLA is still a challenge as the filter wavelength should be adapted to the scale of the event but should also be able to remove properly the mesoscale signal.

The mean regression models based on MOG2D outputs have a lower error (3hPa error), which is explained by the intrinsic definition of the model which only contains the ocean response to wind and pressure forcing. The residual error is likely due to ECMWF forcing errors and to the barotropic model errors. The MOG2D dynamical approach has also pointed out the importance of the spatial variability of the regression models during extreme events, similarly to previous studies for normal meteorological conditions.

Is should be noted that following the Saffir-Simpson scale, each hurricane/depression category is defined within a pressure increment of 15/25hPa, thus observations with accuracy of 5hPa (3hPa for model) or less are valuable particularly when few other sources of data are available, ie. during highly extreme conditions. During ETDs an increase accuracy could be achieved either by tuning the along-track filtering process of the altimeter SLA, or by improving model sea level simulation using better mesh, forcing or model physic.

For TCs’ cases, some statistical regression analysis between the new SLA and the pressure signal could be done in a future work, while considering any accurate SLP from a regional atmospheric model if available, and perform the analysis over several years in order to increase the number of samples.

## Figures and Tables

**Table 1.** Standard deviation (m for SWH and Range, dB for Sigma0) of the 1-Hz measurements for Envisat and Jason-1 for the Ku and C or S bands for standard conditions (2-m SWH and 11-dB backscatter coefficient or sigma0).

	**Envisat**		**Jason-1**	

	**Ku band**	**S band**	**Ku band**	**C band**

**SWH**	0.11	0.42	0.12	0.3
**Range**	0.02	0.07	0.016	0.04
**SIGMA-0**	0.03	0.06	0.02	0.03

**Table 2.** SLP-SLA regression models for 2003 extra-tropical depressions.

**Ocean**	**A (hPa/cm) with 95% confidence level**	**B (hPa)**	**Correlation**	**Nb of samples**	**Error on 2004 rms/mean (hPa)**

**North Atlantic**	−0.796 ± −0.00011	−172.89	−0.83	6962	5.25/−0.4
**North Pacific**	−0.77 ± −0.00011	−173	−0.84	6654	5.2/−0.3
**Indian**	−0.817 ± −0.00008	−175.35	−0.88	6810	5.16/−1.2

**Table 3.** Mean correlation coefficient between the SLP, and percentage of cases for which this correlation is lower than 0.8, for each ocean basin. The upper value is for the Jason/QSCAT correlation, the lower one for Jason/ECMWF.

	**N.Atlantic**	**N.Pacific**	**Indian**

**Mean correlation coefficient**	0.96	0.95	0.96
	0.96	0.94	0.96

**% of correlation coefficient < 0.8**	6.7	8.5	11.8
	8.1	6.4	10.8

**Table 4.** SLP-MOG2D Sea level regression models for 2003 extra-tropical depressions.

**Ocean**	**A (hPa/cm) with 95% confidence level**	**B (hPa)**	**Correlation**	**Nb of samples**	**Error on 2004 (hPa)**

**North Atlantic**	−1.13 ± −0.00005	−2.7	−0.91	7447	3.57/−0.34
**North Pacific**	−1.07 ± −0.00003	−0.95	−0.94	7407	3.45/0.15
**Indian**	−1.2 ± −0.00007	−4.66	−0.87	8109	4.77/−0.81
